# Supplementary material for: Mimicking prophage induction in the body: induction in the lab with pH gradients
Source: PeerJ. 2020 Aug 31;8:e9718. doi: 10.7717/peerj.9718 (PMC7469935; doi:10.7717/peerj.9718)
Supplement: Table S1 [file peerj-08-9718-s001.docx]

**Supplemental Table S1.** Predicted prophages and primer sequences used for identification.

| **Strain** | **Predicted Prophage** | **Primer Sequences** | **Amplicon Length (bp)** |
| --- | --- | --- | --- |
| UMB0149 | N46 Cat2 | TGAACAGGAAGCTGTGATGC & AATTCATAAGCGGCAGTTGG | 324 |
|  | N47 Cat2 | CATGCAGATCACCAACAACC & CGGCAATAGTGCATATGGTG | 310 |
|  | N77 Cat2 | AATCGTGTCGCCAAGGTTAG & TGATACAGAAGCGCAACGTC | 271 |
|  | N97 Cat2 | AACGATCCGTATTCGTCCTG & CATGGTCAATGCCTCTGATG | 309 |
|  | N118 Cat2 | TGCTGTGAACCAAGTCGAAC & TTCAGATGCGCGATAACAAC | 289 |
|  | N7 Cat 5 | CACTGTCATTACCGGACGTG & TAGCGGTCTGGTGCAGTATG | 340 |
|  | N10 Cat5 | AACCGCGTATAGCCAACATC & ACGGTCGATGCCATTAACTC | 287 |
|  | N24 Cat5 | GTGGACATGTTGCTCACACC & GCTCGGTGACTTCCTGAGAC | 306 |
|  | N58 Cat5 | TGCATCGAGGAACAACTGAC & ACGTTCGCCATCATCCTTAG | 287 |
|  | N60 Cat5 | TCGATAGTCTCCACGCACTG & CTCCAGTTGACCGGTTCTTC | 340 |
| UMB527 | N35 Cat2 | GGTTGTTCTCTGCTGCTTCC & TGCCAGTGAGCAAGATTGAC | 309 |
|  | N69 Cat2 | TGGCCGGAGATACAACCTAC & AGTTACGCTGCTCACCATCC | 280 |
|  | N101 Cat2 | TCATGCCAAGCTGCTGATAG & ATAACTGGCGTTACCGCATC | 345 |
|  | N113 Cat2 | CAGCTTCCTGCCTTATCTCG & CTGCAGATTGATGGACATGC | 334 |
|  | N5 Cat4 | GCGTTCGCGAGAAGATTAAC & CAAGCTTCAGCTCGTCAGTG | 304 |
|  | N1 Cat5 | TCAACCATGCGTTACAGCTC & AACTCCGACAGGTCCACATC | 273 |
|  | N12 Cat5 | TAACATTGAGCAGGCTGACG & ACCGTCCTCTGCCACATAAG | 307 |
|  | N20 Cat5 | TCAGGTAATCCATCGCCTTC & ATTGTAACGACAGGCCGAAC | 327 |
|  | N28 Cat5 | AGACTGGCCACTTCGGTATG & GGCTGAAGGAGGTCTCTGTG | 319 |
|  | N32 Cat5 | AACCACCTCGCAGTATCTGG & GGCCATGTTGTTGCTGTATG | 346 |
|  | N45 Cat5 | TGCTGTGAACCAAGTCGAAC & TTCAGATGCGCGATAACAAC | 289 |
|  | N55 Cat5 | TGGTTCACCGAGACCTAACC & AGAGGACGACATGAGCAAGC | 280 |
|  | N60 Cat5 | AGTACACGCTGGATGGTTCC & TGCGCTGAACTATCTGGATG | 309 |
| UMB906 | N31 Cat2 | AGGTGGTCTCCACCAGTGAC & ATGATGAGCGTGATTGATGC | 310 |
|  | N1 Cat5 | CACACGGTTGACCAGAACAC & ATCACCAGAGATGCCTCCAC | 306 |
|  | N5 Cat5 | ATGGTGAGCCGGTCTATCTG & CCACGATCCATCCTTCAGTC | 318 |
|  | N9 Cat5-1 | GCGAAGACTGGAAGCGTATC & CCTTGTCGGTGAAGAGGAAG | 339 |
|  | N9 Cat5-2 | ATATACGCCTGCTCCACACC & GGTGTTAATGATGGCCTTGG | 320 |
|  | N13 Cat5 | TCAGCGATTACGATGTCAGC & TGATAGACCACGGCACTACG | 305 |
|  | N16 Cat5 | TGAACTCGTACGCCAGAGTG & GCTGCATAACTTCGCTCCTC | 323 |
| UMB923 | N16 Cat2 | CAGCCGCATGAATATGTGAC & CGATGTGCAATACCACGAAC | 323 |
|  | N3 Cat4 | AGGCACAATAGGTGCAATCC & ATTACATCGCCGTCGTAAGC | 270 |
|  | N1 Cat5-1 | CCGGATCACCTTCTTCTGAG & AGATGGCTAACGATCCATGC | 297 |
|  | N1 Cat5-2 | TACAGACCGCGATACTGCAC & TCCGGTTACTCTGTGGTTCC | 293 |
|  | N4 Cat5 | CAACCGGTATGACTGTGACG & CGCTGACTCTCCGGATTAAC | 275 |
| UMB934 | N52 Cat2 | GTCTTCACGAAGGAGGCAAC & GAAGGAGTCGAAGCCACATC | 346 |
|  | N61 Cat2 | TCTGATGGCAGCAATAGTGG & GCCTGCATGTCTCCTCCTAC | 338 |
|  | N72 Cat2 | AGGTGACACCAGCCGTTAAG & ACATTCAGGATCGGCAAGAC | 311 |
|  | N84 Cat2 | ACTTCTGGTCCGGTAACGTG & ACACACCGTTGATGATCTGC | 281 |
|  | N1 Cat5 | AATGCGGCAACCATAGACTC & CGTGATGAATACACGGCAAG | 294 |
|  | N5 Cat5 | AGGTTGTGGTGCTGGTTCTC & CATAGCCTGCTTCTCCTTGC | 312 |
|  | N9 Cat5 | CCTCTTCGTCGAACTTACGC & AACGACACACTTGCTTCACG | 334 |
|  | N25 Cat5 | GGTAGCCGCTGAGATTGAAG & ACGGCAGATGGTCCAGATAC | 349 |
|  | N39 Cat5 | AGAACTGACACAGGCCGAAG & GTTAACTTCCGGAGCCACAC | 297 |
| UMB1160 | N32 Cat1 | TGTCACAGGCGTCAGAAGAC & AGTATGCCAACCGCACTACC | 289 |
|  | N36 Cat2 | ATCGCGGTGTATTCAGAACC & CAGCACAGCTGTAGCCTGAC | 274 |
|  | N2 Cat4 | TGATGATACAGGCGAACTGC & ACGTTGCAATAACGGTAGCC | 308 |
|  | N8 Cat5 | CAGCGATGATGACGATTGAC & GTCAGTTCTTCCGCCTTGAG | 322 |
|  | N12 Cat5 | AGCCGGTAATGACCTTGATG & ACGTGACAGCACAGAGATGG | 274 |
|  | N20 Cat5 | AACATGGACTCCTGGTGGAC & AACCGTTACATTCCGTCAGC | 319 |
|  | N21 Cat5 | CACACGGCTGACCATAACAC & TCACCAGAGATGCTTCAACG | 305 |
| UMB1335 | N11 Cat4 | GCCTGGCGTGTCCATATC & AGCCGCTGATGCTTGAAC | 350 |
