# Supplementary material for: Mimicking prophage induction in the body: induction in the lab with pH gradients
Source: PeerJ. 2020 Aug 31;8:e9718. doi: 10.7717/peerj.9718 (PMC7469935; doi:10.7717/peerj.9718)
Supplement: Table S2 [file peerj-08-9718-s002.docx]

**Supplemental Table S2.** Thermocycler conditions for phage amplification.

| **Step** | **Temperature (°C)** | **Time** |
| --- | --- | --- |
| 1. Initial denaturation | 95 | 3 m |
| 2. Repeat 30x |  |  |
| Denaturation | 95 | 30 s |
| Annealing | 55 | 30 s |
| Extension | 72 | 30 s |
| 3. Final extension | 72°C | 5 m |
